# Risk for Cardiovascular Disease and One-Year Mortality in Patients With Chronic Obstructive Pulmonary Disease and Obstructive Sleep Apnea Syndrome Overlap Syndrome

**DOI:** 10.3389/fphar.2021.767982

**Published:** 2021-10-26

**Authors:** Manyun Tang, Yidan Wang, Mengjie Wang, Rui Tong, Tao Shi

**Affiliations:** ^1^ Department of Cardiovascular Medicine, The First Affiliated Hospital of Xi’an Jiaotong University, Xi’an, China; ^2^ Department of Geriatric Endocrinology, The First Affiliated Hospital of Xi’an Jiaotong University, Xi’an, China; ^3^ Department of Cardiovascular Surgery, The First Affiliated Hospital of Xi’an Jiaotong University, Xi’an, China

**Keywords:** cardiopulmonary diseases, pulmonary hypertension, chronic obstructive pulmonary disease, obstructive sleep apnea syndrome, overlap syndrome, prognosis

## Abstract

**Background:** Patients with chronic obstructive pulmonary disease (COPD) and obstructive sleep apnea (OSAS) overlap syndrome (OS) are thought to be at increased risk for cardiovascular diseases.

**Objective:** To evaluate the burden of cardiovascular diseases and long-term outcomes in patients with OS.

**Methods:** This was a retrospective cohort study. The prevalence of cardiovascular diseases and 1-year mortality were compared among patients diagnosed with OS (OS group), COPD alone (COPD group) and OSAS alone (OSAS group), and Cox proportional hazards models were used to assess independent risk factors for all-cause mortality.

**Results:** Overall, patients with OS were at higher risk for pulmonary hypertension (PH), heart failure and all-cause mortality than patients with COPD or OSAS (all *p* < 0.05). In multivariate Cox regression analysis, the Charlson comorbidity index (CCI) score [adjusted hazard ratio (aHR): 1.273 (1.050–1.543); *p* = 0.014], hypertension [aHR: 2.006 (1.005–4.004); *p* = 0.048], pulmonary thromboembolism (PTE) [aHR: 4.774 (1.335–17.079); *p* = 0.016] and heart failure [aHR: 3.067 (1.521–6.185); *p* = 0.002] were found to be independent risk factors for 1-year all-cause mortality.

**Conclusion:** Patients with OS had an increased risk for cardiovascular diseases and 1-year mortality. More efforts are needed to identify the causal relationship between OS and cardiovascular diseases, promoting risk stratification and the management of these patients.

## Introduction

Chronic obstructive pulmonary disease (COPD) and obstructive sleep apnea syndrome (OSAS) are two common chronic diseases with increasing incidence worldwide and impose a heavy burden on the healthcare system (Lévy et al., 2015; Rabe and Watz, 2017). Moreover, both COPD and OSAS are considered to be risk factors for cardiovascular diseases (Tang et al., 2021). Common cardiovascular diseases associated with COPD and OSAS include hypertension, stroke, heart failure, atrial fibrillation, and coronary heart disease (CHD) (McNicholas et al., 2007; Macnee et al., 2008). The underlying mechanisms are multifactorial, including hypoxia, hypercapnia, systemic inflammation, oxidative stress, increased sympathetic nervous system activity, and endothelial dysfunction (Wolf et al., 2007; Macnee et al., 2008; Kohler and Stradling, 2010; Maclay and MacNee, 2013). COPD is a major risk factor for cardiovascular morbidity and mortality (Sin and Man, 2005), and evidence from a cohort study also suggests higher cardiovascular-related and all-cause mortality in patients with severe OSAS (Young et al., 2008).

Overlap syndrome (OS) is defined as coexisting COPD and OSAS in a single patient (Flenley, 1985). Studies have shown that patients with OS develop greater nocturnal oxygen desaturation and more severe hypercapnia, systemic inflammation, and endothelial dysfunction than patients with OSAS or COPD alone (Chaouat et al., 1995; McNicholas, 2017; Tang et al., 2021). All these findings suggest that patients with OS are at greater risk of cardiovascular diseases; however, there are few studies on cardiovascular morbidity and mortality in these patients, and the underlying risk factors are still unclear. Therefore, this study was conducted to evaluate the risk of cardiovascular diseases and 1-year mortality in OS patients and provide more real-world data on the burden of cardiovascular diseases in this cohort.

## Methods

### Ethical Approval and Consent

This retrospective cohort study was performed in the Department of Cardiovascular Medicine and Respiratory Medicine of The First Affiliated Hospital of Xi’an Jiaotong University. The study was conducted in accordance with the Declaration of Helsinki and approved by the Ethics Committee of The First Affiliated Hospital of Xi’an Jiaotong University (No. XJTU1AF2020LSK-187). Written informed consents were obtained from the patients or their family members prior to the use of their anonymized clinical data.

### Patients

Patients with a diagnosis of COPD or OSAS and discharged between July 2014 and July 2020 were initially searched in the Biobank of The First Affiliated Hospital of Xi’an Jiaotong University. All collected participants had to undergo screening. The exclusion criteria were: age <18 or >80 years old, loss to follow-up, lack of clinical data, previous upper airway surgery for OSAS, preexisting serious comorbidities or life expectancy less than 1 year, severe COPD with noninvasive or invasive respiratory ventilator use and unwillingness to participate in the study. Then, the consecutively enrolled patients were divided into three groups: (1) the OS group, (2) the COPD group, and (3) the OSAS group. OS was defined as coexisting COPD and OSAS in one patient. The two control groups were pair-matched with the OS group based on age and sex at a 1:2 ratio.

### Clinical Data Collection

This study was observational, reflected routine practice and did not interfere with medical management. Patients who met the criteria were consecutively enrolled, and their clinical data were collected from the Biobank of The First Affiliated Hospital of Xi’an Jiaotong University by designated investigators and recorded on dedicated electronic forms.

The patients’ anonymous clinical data included baseline demographics [age, sex, body mass index (BMI)], medical history [hypertension, diabetes, hyperlipidemia, stroke, pulmonary thromboembolism (PTE) and smoking], laboratory examination (complete blood counts, biochemical tests, coagulation function, and arterial blood gas analysis), pulmonary function tests, full-night polysomnography and treatments [CPAP and medications (ACEIs/ARBs, β-blockers, antisterone, nifedipine, diuretic, and aspirin)]. The screening for the diagnosis of diseases was performed according to the International Classification of Diseases-10.

### Outcomes and Follow-Up

We followed all enrolled patients from the index date to the end of 1 year. The primary endpoint was all-cause death, and survival status were obtained from outpatient records or telephone contact with the patients or their relatives. The secondary endpoint was the prevalence of four specific cardiovascular diseases, including pulmonary hypertension (PH), heart failure, arrhythmias, and CHD.

### Statistical Analysis

Continuous variables were described as the mean and standard deviation (SD) and were tested for normal distribution by the Kolmogorov-Smirnov test. Analysis of variance with a post hoc test was used to compare continuous variables that satisfied normal distribution, and the Kruskal-Wallis test was used to compare continuous variables that did not satisfy normal distribution. Categorical variables were summarized as counts and percentages and compared by the chi-square test. As three comparisons of baseline characteristics and the incidence of cardiovascular events were performed, a *p*-value threshold of <0.017 was used for the descriptive analysis after applying Bonferroni correction. To evaluate the risk factors for all-cause mortality between OS, COPD, and OSAS patients during follow-up, univariate and multivariate Cox proportional hazards models were used to assess HR and 95% confidence interval (CI). The multivariable model was adjusted for covariates (BMI, the Charlson comorbidity index (CCI), hypoxemia, hypercapnia, respiratory failure, hypertension, diabetes, hyperlipidemia, stroke, PTE, heart failure, arrhythmia, and CHD). Furthermore, the 1-year mortality of the three groups was evaluated using the Kaplan-Meier method and compared by the use of log-rank tests. All data processing and statistical analyses were performed using the SPSS 26.0 statistical package, and a *p*-value < 0.05 was considered indicative of statistical significance.

## Results

### Baseline Characteristics

**FIGURE 1:**
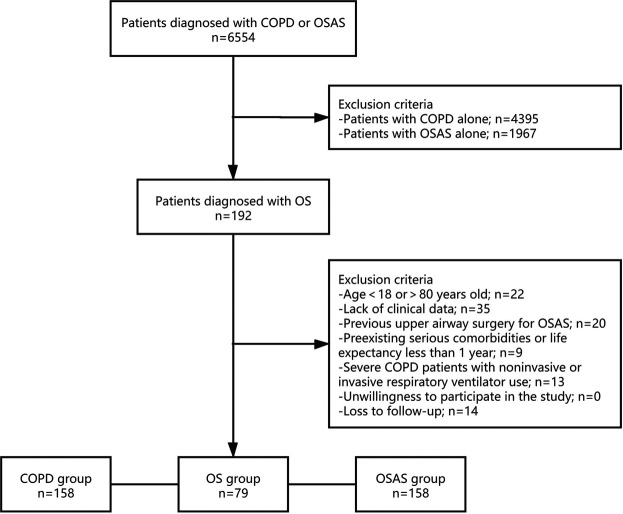
Study Protocol. Flowchart of the selection process of the current study. OS, overlap syndrome; COPD, chronic obstructive pulmonary disease; OSAS, obstructive sleep apnea syndrome.

Among the 6,554 patients initially collected from the biobank according to the diagnosis of COPD or OSAS, 4,587 had COPD, and 2,159 had OSAS. There were 192 patients suffering from COPD and OSAS overlap syndrome. Overall, 79 patients were enrolled in the OS group after screening (Figure 1). The rate of loss to follow-up was 7.3%. The clinical characteristics of the patients in the OS group, COPD group and OSAS group are described in Table 1.

**TABLE 1:** Baseline characteristics of all patients.

	All	OS group	COPD group	OSAS group	*p* Value	*p* Value OS and COPD	*p* Value OS and OSAS
	*n* = 395	*n* = 79	*n* = 158	*n* = 158			
Age (years)	60.6 ± 9.5	60.7 ± 9.6	60.6 ± 9.5	60.6 ± 9.6	0.993		
Male, n (%)	275 (69.6)	55 (69.6)	110 (69.6)	110 (69.6)	1.000		
BMI (kg/m^2^)	25.0 ± 5.1	26.5 ± 2.3	24.4 ± 7.1	24.8 ± 3.4	<0.001	<0.001	<0.001
Smoker, n (%)	92 (23.3)	23 (29.1)	35 (22.2)	34 (21.5)	0.388		
Smoking index	717.6 ± 412.0	863.5 ± 487.8	622.6 ± 293.5	716.9 ± 442.2	0.130		
Laboratory data							
PO_2_ (mmHg)	72.0 ± 15.3	60.2 ± 12.7	74.5 ± 16.2	75.4 ± 12.5	<0.001	<0.001	<0.001
PCO_2_ (mmHg)	48.1 ± 11.2	57.1 ± 12.2	47.5 ± 10.7	44.1 ± 8.2	<0.001	<0.001	<0.001
SaO_2_ (%)	91.2 ± 7.2	86.5 ± 9.4	92.8 ± 5.0	92.0 ± 6.9	<0.001	<0.001	<0.001
D-dimer (mg/L)	1.0 ± 1.9	1.3 ± 1.7	1.2 ± 2.2	0.8 ± 1.6	<0.001	0.497	<0.001
Fibrinogen (g/L)	3.5 ± 1.3	3.5 ± 1.3	3.8 ± 1.4	3.1 ± 0.9	<0.001	0.226	0.033
Hemoglobin (g/L)	144.3 ± 21.7	149.7 ± 25.9	142.4 ± 21.0	143.6 ± 19.7	0.053	0.051	0.118
CRP (mg/L)	9.8 ± 23.2	14.8 ± 30.3	11.2 ± 25.0	5.8 ± 15.3	<0.001	0.373	<0.001
Cholesterol (mmol/L)	4.0 ± 1.1	4.0 ± 1.4	4.0 ± 1.0	4.0 ± 1.2	0.459		
Triglyceride (mmol/L)	1.6 ± 1.0	1.7 ± 1.0	1.2 ± 0.3	1.8 ± 1.2	<0.001	<0.001	0.559
LDL (mmol/L)	2.2 ± 0.6	2.3 ± 0.7	2.0 ± 0.3	2.4 ± 0.8	<0.001	<0.001	0.440
BNP (pg/ml)	778.9 ± 2138.5	1181.8 ± 2286.2	937.7 ± 2729.5	418.6 ± 1091.8	<0.001	<0.001	<0.001
cTnT (ng/ml)	0.05 ± 0.24	0.03 ± 0.02	0.10 ± 0.38	0.02 ± 0.05	<0.001	0.700	0.001
CK (U/L)	101.8 ± 207.7	76.8 ± 105.4	91.7 ± 139.1	124.2 ± 286.9	<0.001	0.202	<0.001
CKMB (U/L)	16.1 ± 19.4	17.3 ± 17.6	14.7 ± 10.0	16.9 ± 26.2	0.764		
LDH (U/L)	245.2 ± 96.7	264.2 ± 80.5	261.5 ± 125.0	219.4 ± 59.1	<0.001	0.302	<0.001
Medications							
ACEI/ARB, n (%)	127 (32.2)	25 (31.6)	26 (16.5)	76 (48.1)	<0.001	0.007	0.016
Beta-blockers, n (%)	109 (27.6)	21 (26.6)	25 (15.8)	61 (38.6)	<0.001	<0.001	0.067
Antisterone, n (%)	90 (22.8)	26 (32.9)	40 (25.3)	24 (15.2)	0.006	0.029	0.002
Nifedipine, n (%)	87 (22.0)	26 (32.9)	24 (15.2)	37 (23.4)	0.007	0.002	0.019
Diuretic, n (%)	118 (29.9)	48 (60.8)	44 (27.8)	26 (16.5)	<0.001	<0.001	<0.001
Aspirin, n (%)	164 (41.5)	29 (36.7)	29 (18.4)	106 (67.1)	<0.001	<0.001	<0.001
Comorbidities							
CCI	3.0 ± 1.5	3.5 ± 1.5	2.9 ± 1.2	2.8 ± 1.6	0.006	0.009	0.005
Hypertension, n (%)	214 (54.2)	56 (70.9)	45 (28.5)	113 (71.5)	<0.001	<0.001	0.919
Diabetes, n (%)	112 (28.4)	30 (38.0)	29 (18.4)	53 (33.5)	0.001	0.001	0.500
Hyperlipidemia, n (%)	22 (5.6)	3 (3.8)	3 (1.9)	16 (10.1)	0.004	0.403	0.091
Stroke, n (%)	70 (17.7)	15 (19.0)	21 (13.3)	34 (21.5)	0.151	0.249	0.650
PTE, n (%)	5 (1.3)	4 (5.1)	1 (0.6)	0 (0)	0.006	0.014	0.012
Hypoxemia, n (%)	319 (80.8)	72 (91.1)	127 (80.4)	120 (75.9)	0.020	0.033	0.005
Hypercapnia, n (%)	96 (24.3)	55 (69.6)	33 (20.9)	8 (5.1)	<0.001	<0.001	<0.001
Respiratory failure, n (%)	56 (14.2)	29 (36.7)	17 (10.8)	10 (6.3)	<0.001	<0.001	<0.001

OS, overlap syndrome; COPD, chronic obstructive pulmonary disease; OSAS, obstructive sleep apnea syndrome; BMI, body mass index; PO2, oxygen partial pressure; PCO2, partial pressure of carbon dioxide; SaO2, oxygen saturation; CRP, C-reactive protein; LDL, low-density lipoprotein; BNP, B-type natriuretic peptide; cTnT, cardiac troponin T; CK, creatine kinase; CKMB, creatine kinase-MB; LDH, lactate dehydrogenase; ACEI, angiotensin-converting enzyme inhibitors; ARB, angiotensin receptor blockers; CCI, Charlson comorbidity index; PTE, pulmonary thromboembolism.

The study population was predominantly male (69.6%), with an average age of 60.6 ± 9.5 years. Patients with OS had significantly lower blood oxygen pressure (PO_2_) and arterial oxygen saturation (SaO_2_) and higher BMI, partial pressure of carbon dioxide (PCO_2_), and serum levels of B-type natriuretic peptide (BNP) than patients with COPD or OSAS alone (all *p* < 0.001). In the OS group, the serum levels of D-dimer, C-reactive protein (CRP), cardiac troponin T (cTnT), and lactate dehydrogenase (LDH) were higher than those in the OSAS group (all *p* < 0.001); and the serum levels of triglyceride and low-density lipoprotein (LDL) were higher than those in the COPD group (all *p* < 0.001).

Regarding medications, antisterone, nifedipine and diuretic were more commonly used in the OS group than in other two groups. There was a higher tendency to use angiotensin-converting enzyme inhibitors (ACEIs)/angiotensin receptor blockers (ARBs), beta-blockers and aspirin in OS patients than in COPD patients (all *p* < 0.001); however, compared with OSAS patients, this tendency was significantly reduced.

Patients with OS had higher CCI scores (*p* = 0.006) and were more likely to concomitantly suffer PTE (*p* = 0.006), hypercapnia (*p* < 0.001), and respiratory failure (*p* < 0.001) than patients with COPD or OSAS alone. More patients had hypertension (*p* < 0.001) and diabetes mellitus (*p* = 0.001) in the OS group than in the COPD group.

### Severity of COPD and OSAS of Patients in Three Groups

Table 2 shows the pulmonary function tests and GOLD classification of patients in OS and COPD group, respectively. No between-group difference in the GOLD classification was observed (all *p* > 0.05), FEVI% in COPD group was lower than that in OS group (40.8 ± 19.8 vs. 44.6 ± 19.4), though not statistically significant.

**TABLE 2:** Pulmonary function tests and GOLD classification.

	All	OS group	COPD group	*p* Value
	*n* = 237	*n* = 79	*n* = 158	
FEV1/FVC ratio (%)	55.2 ± 15.7	58.7 ± 16.8	53.4 ± 14.9	0.015
FEV1% predicted	42.0 ± 19.7	44.6 ± 19.4	40.8 ± 19.8	0.109
Severity of COPD, n (%)				
GOLD 1	14 (5.9)	4 (5.1)	10 (6.3)	0.779
GOLD 2	67 (28.3)	27 (34.2)	40 (25.3)	0.153
GOLD 3	74 (31.2)	24 (30.4)	50 (31.6)	0.843
GOLD 4	82 (34.6)	24 (30.4)	58 (36.7)	0.334

FEV1, orced expiratory volume in 1 s; FVC, forced vital capacity; GOLD, Global Initiative for Chronic Obstructive Lung Disease; other abbreviations as in Table 1.

The baseline polysomnography data and treatment of patients in OS group and OSAS group were shown in Table 3. In the sleep testing study, the apnea-hypopnea index (AHI) was higher in patients with OSAS than in those with OS (26.5 ± 16.6 vs. 25.1 ± 15.4; *p* = 0.521), there was no obvious difference in the distribution of severity of OSAS in two groups. However, patients in the OS group were more likely to receive CPAP therapy than those in the OSAS group (57.0 vs. 15.8%; *p* < 0.001).

**TABLE 3:** Baseline polysomnography data and treatment.

	All	OS group	OSAS group	*p* Value
	*n* = 237	*n* = 79	*n* = 158	
AHI	26.0 ± 16.2	25.1 ± 15.4	26.5 ± 16.6	0.521
Severity of OSAS, n (%)				
Mild	69 (29.1)	21 (26.6)	48 (30.4)	0.544
Moderate	81 (34.2)	35 (44.3)	46 (29.1)	0.020
Severe	87 (36.7)	23 (29.1)	64 (40.5)	0.086
CPAP, n (%)	70 (29.5)	45 (57.0)	25 (15.8)	<0.001

AHI = apnea–hypopnea index; CPAP = continuous positive airway pressure; other abbreviations as in Table 1.

### Prevalence of Cardiovascular Events

Table 4 shows the incidence of five specific cardiovascular diseases—CHD, arrhythmias, heart failure, stroke and PH—in the OS group, COPD group and OSAS group. There was a significant increase in the incidence of heart failure (35.4 vs. 8.9% and 0.6%; *p* < 0.001) and PH (36.7 vs. 17.1% and 4.4%; *p* < 0.001) in the OS group compared with the COPD group or OSAS group. Patients with OS had a higher prevalence of CHD than patients with OSAS (30.4 vs. 9.5%; *p* < 0.001). The prevalence of stroke and arrhythmias did not differ significantly among the three groups, whereas more patients in the OS group had atrial fibrillation than those in the COPD group (13.9 vs. 4.4% and 6.8%; *p* = 0.018).

**TABLE 4:** Incidence of cardiovascular diseases in study patients.

	All	OS group	COPD group	OSAS group	*p* value	*p* value OS and COPD	*p* value OS and OSAS
	*n* = 395	*n* = 79	*n* = 158	*n* = 158			
CHD, n (%)	97 (24.6)	24 (30.4)	15 (9.5)	58 (36.7)	<0.001	<0.001	0.334
Arrhythmia, n (%)	58 (14.7)	18 (22.8)	19 (12.0)	21 (13.3)	0.072	0.031	0.063
Conduction block, n (%)	13 (3.3)	5 (6.3)	4 (2.5)	4 (2.5)	0.296	0.280	0.280
Ventricular arrhythmias, n (%)	9 (2.3)	2 (2.5)	4 (2.5)	9 (2.3)	1.000	1.000	1.000
Atrial arrhythmia, n (%)	36 (9.1)	11 (13.9)	11 (7.0)	14 (8.9)	0.212	0.082	0.232
AF, n (%)	27 (6.8)	11 (13.9)	7 (4.4)	9 (6.8)	0.018	0.009	0.032
Heart failure, n (%)	43 (10.9)	28 (35.4)	14 (8.9)	1 (0.6)	<0.001	<0.001	<0.001
Stroke, n (%)	70 (17.7)	15 (19.0)	21 (13.3)	34 (21.5)	0.151	0.249	0.650
PH, n (%)	64 (16.2)	29 (36.71)	28 (17.1)	7 (4.4)	<0.001	0.001	<0.001
Mild	33 (51.6)	14 (48.3)	15 (53.6)	4 (57.1)			
Moderate	20 (31.2)	11 (37.9)	7 (25.0)	2 (28.6)			
Severe	11 (17.2)	4 (13.8)	6 (21.4)	1 (14.3)			

CHD, coronary heart disease; AF, atrial fibrillation; PH, pulmonary hypertension; other abbreviations as in Table 1.

### Risk Factors for All-Cause Mortality

**FIGURE 2:**
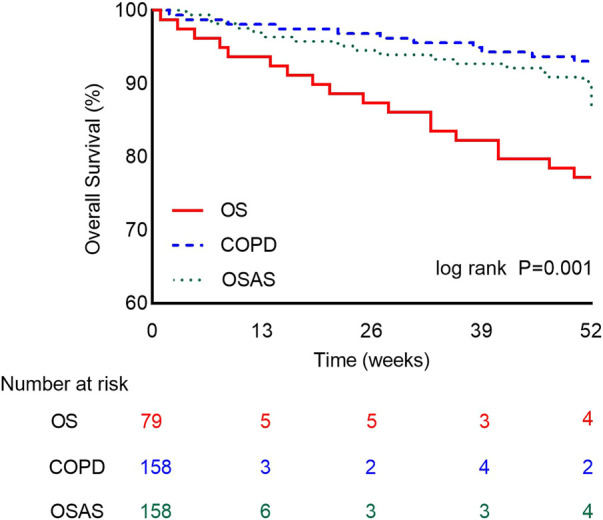
The One-Year Kaplan-Meier Survival Curves. Survival rate between patients in the OS group (red line), COPD group (blue line), and OSAS group (green line). The difference is statistically significant. (Log-rank test = 14.128, *p* = 0.001).

The primary outcome, all-cause death during the 1-year follow-up, occurred in 21.5% (17/79) of patients with OS, 7.0% (11/158) of patients with COPD and 10.1% (16/158) of patients with OSAS. The 1-year mortality was significantly higher in the OS group than in the other two groups (*p* = 0.003). The overall survival rate was lower in the OS group than in the COPD or OSAS group (Figure 2). In univariate Cox regression analysis, OS (HR: 2.892 [1.593–5.252], *p* < 0.001), the CCI score (HR: 1.277 [1.057–1.542], *p* = 0.011), hypercapnia (HR: 1.968 [1.077–3.597], *p* = 0.028), respiratory failure (HR: 2.370 [1.224–4.590], *p* = 0.010), hypertension (HR: 2.418 [1.249–4.683], *p* = 0.009), stroke (HR: 2.199 [1.169–4.134], *p* = 0.014), PTE (HR: 5.907 [1.828–19.083], *p* = 0.003), and heart failure (HR: 2.993 [1.516–5.907], *p* = 0.002) were associated with all-cause mortality during the 1-year follow-up. After adjusting for OS, BMI, the CCI score, smoking status, hypoxemia, hypercapnia, respiratory failure, diabetes, hyperlipidemia, stroke, PTE, PH, heart failure, arrhythmia, and CHD in multivariate Cox regression analysis, the CCI score (adjusted HR [aHR]: 1.273 [1.050–1.543]; *p* = 0.014), hypertension (aHR: 2.006 [1.005–4.004]; *p* = 0.048), PTE (aHR: 4.774 [1.335–17.079]; *p* = 0.016) and heart failure (aHR: 3.067 [1.521–6.185]; *p* = 0.002) were identified as independent predictors of all-cause mortality during the 1-year follow-up (Table 5).

**TABLE 5:** Results of univariate and multivariate Cox regression analysis.

	Univariate analysis			Multivariate analysis		
Variables	Curde HR	95%CI	*p* Value	Adjusted HR*	95%CI	*p* Value
OS	2.892	1.593–5.252	<0.001			
BMI	0.987	0.923–1.056	0.709			
CCI	1.277	1.057–1.542	0.011	1.273	1.050–1.543	0.014
smoker	0.697	0.324–1.496	0.354			
hyoxemia	1.132	0.527–2.430	0.751			
hypercapnia	1.968	1.077–3.597	0.028			
Respiratory failure	2.370	1.224–4.590	0.010			
hypertension	2.418	1.249–4.683	0.009	2.006	1.005–4.004	0.048
diabetes	1.215	0.628–2.353	0.563			
hyperlipidemia	1.434	0.445–4.628	0.546			
stroke	2.199	1.169–4.134	0.014			
PTE	5.907	1.828–19.083	0.003	4.774	1.335–17.079	0.016
PH	1.773	0.899–3.500	0.099			
heart failure	2.993	1.516–5.907	0.002	3.067	1.521–6.185	0.002
arrhythmia	1.752	0.905–3.393	0.096			
CHD	1.237	0.649–2.357	0.517			

*Multivariable model adjusts forOS, BMI, CCI, smoker, hyoxemia, hypercapnia, respiratory failure, diabetes, hyperlipidemia, stroke, PTE, PH, heart failure, arrhythmia, and CHD. HR, hazard ratio; CI, confidence interval; other abbreviations as in Tables 1, 4.

## Discussion

In this study, OS was observed in 4.2% (192/4,587) of COPD patients and 8.9% (192/2,159) of OSAS patients. The prevalence of PH and heart failure was significantly higher in the OS group than in the COPD or OSAS group. Moreover, the OS group had a higher proportion of patients with CHD than the COPD group, suggesting a heavier cardiovascular burden in patients with OS. Moreover, patients with OS had a higher 1-year all-cause mortality than patients with COPD or OSAS alone. After adjusting for confounders, the rate of all-cause death was identified to be independently associated with the CCI score, hypertension, PTE and heart failure. To our knowledge, this is the first study to evaluate the burden of cardiovascular diseases and prognosis and search for risk factors among OS, COPD and OSAS populations.

The mechanisms that cause cardiovascular diseases in patients with COPD or OSAS are multifactorial, including hypoxia, systemic inflammation, oxidative stress, endothelial dysfunction and a hypercoagulable state (McNicholas et al., 2007; Wolf et al., 2007; Macnee et al., 2008; Kohler and Stradling, 2010; Maclay and MacNee, 2013; Cowie et al., 2021). As the severity of COPD or OSAS increases, cardiovascular diseases become more prevalent (Curkendall et al., 2006; Mills et al., 2008). In addition, in this study, there was a fact that patients with OS, of whom the FEV1% was higher and the AHI was lower than patients with COPD and OSAS, respectively, developed more severe oxygen desaturation and hypercapnia, suggesting a synergistic effect of COPD and OSA in one patient. This may, to some extent, explain why patients with OS are at greater risk of cardiovascular disease than patients with COPD or OSAS alone.

Hypoxemia has been identified as a risk factor for the development of cardiovascular disease in many studies because it can promote the release of reactive oxygen species, induce oxidative stress, and impair endothelial function (Bosc et al., 2010; Dewan et al., 2015). Nocturnal hypoxemia in OS patients has been confirmed by the results of sleep monitoring in many previous studies, and it usually manifests as decreased mean and the lowest SaO2 and increased sleep time with SaO2 < 90% (Chaouat et al., 1995; Shiina et al., 2012). Our study indicated that patients with OS had more severe hypoxia. In this study, we collected and further evaluated the patients’ arterial blood gas analysis in three groups, finding that daytime hypoxemia, and hypercapnia were more common in OS patients. As clinical evidence has established that the majority of OSAS patients are eucapnic while conscious, the detection of daytime hypercapnia suggested a further impairment of the respiratory system in OSAS patients who had COPD. Moreover, evidence from cell culture studies, animal models, and controlled studies in humans has demonstrated that intermittent hypoxia can promote oxidative stress (Takabatake et al., 2000; Zhan et al., 2005; Mills et al., 2008; Pialoux et al., 2009), which is another risk factor for cardiovascular diseases. This more pronounced hypoxemia and hypercapnia might be one of the causes of increased cardiovascular morbidity and mortality.

Patients with COPD, OSAS, or OS always have an active status of systemic inflammation, which is a key factor that predisposes these individuals to cardiovascular diseases. C-reactive protein, an independent predictor of cardiovascular disease and an important serum marker of inflammation (Rutter et al., 2004), was significantly increased in the OS group, indicating an accelerated inflammatory process in this cohort. In addition, we found that patients in the OS group had a higher BMI. This finding was consistent with a previous study in that obesity can also induce systemic inflammation by upregulating the expression of systemic inflammatory mediators (Trayhurn and Wood, 2004).

In addition to hypoxia and inflammation, abnormal lipid metabolism also contributes to cardiovascular disease. An important interaction between hypoxia and lipids is assumed to exist in the development of atherosclerosis (Li et al., 1985). In an animal study, chronic intermittent hypoxia combined with a high-fat diet was found to promote the progression of atherosclerosis in male C57BL/6 mice, whereas hypoxia or a high-fat diet alone did not (Savransky et al., 2007). Evidence from a case-control study carried out by Barceló A also identified the abnormal lipid peroxidation in patients with sleep apnoea (Barceló et al., 2000). In our study, we found that abnormal lipid metabolism at baseline was more common in COPD patients who also had OSA and was characterized by higher serum levels of triglycerides and low-density lipoprotein (LDL). This may explain, to some extent, why OS patients had a higher incidence of coronary heart disease than COPD patients in our study.

COPD and OSAS have been reported to be correlated with hypertension, stroke, arrhythmia, heart failure and ischemic heart disease. Voulgaris et al. reported an increased risk for cardiovascular disease in OS patients by using the Framingham Risk Score (FRS) and Systematic COronary Risk Evaluation (SCORE) scoring models (Voulgaris et al., 2019), but specific changes in the incidence of each cardiovascular disease were not discussed. In this study, significantly higher incidence of heart failure and PH was observed in the OS group, and CHD was also more common in the OS group than in the COPD group. As in this study, most patients who had OSAS were diagnosed during the treatment of CHD, increasing the proportion of CHD in the OSAS population, so no significant difference in the prevalence of CHD was observed between the OS group and the OSAS group.

PH, a common complication of COPD, is the result of the remodeling of pulmonary arteries. Pulmonary vascular remodeling in COPD patients is considered to be caused by elevated pulmonary artery pressure due to a combination of hypoxia, inflammation, and impairment of endothelial function (Barberà and Blanco, 2009). There are also some studies exploring the relationship between OSAS and PH, confirming that the OSAS is associated with mild PH and this may be due to a combination of precapillary and postcapillary factors, such as pulmonary arteriolar remodeling, hyperreactivity to hypoxia, systemic inflammation and left atrial enlargement (Kessler et al., 1996; Sajkov and McEvoy, 2009). In this study, we found that in OS patients, those who had more severe hypoxia and systemic inflammation than patients with OSAS or COPD alone, the prevalence of PH increased significantly, suggesting that physicians should pay more attention to those COPD patients who already have OSAS or OSAS patients who have COPD.

During the 1-year follow-up, there were 17 (21.5%) all-cause deaths in the OS group, 11 (7.0%) all-cause deaths in the COPD group, and 16 (10.1%) all-cause deaths in the OSAS group, and a significantly higher mortality rate was found in the OS group. After adjusting for confounding factors, the CCI score, hypertension, PTE and heart failure were assumed to be independent risk factors for 1-year mortality in this population, suggesting a heavier burden of cardiovascular disease in this population. The causal relationship between OS and cardiovascular diseases is still unclear. This study provides an early recognition of the burden of cardiovascular disease in OS patients. We hope that it can promote timely aggressive management of these patients, including lifestyle changes and treatment targeting the CCI score, hypertension, PTE and heart failure, to improve these patients’ long-term outcomes.

There are some limitations should be highlighted. First, this was a single-center study, and the study sample was small. Second, given the retrospective nature of the study, some clinical information, such as Holter and telemetry monitoring, was not available, which limited our assessment of recurrent arrhythmias. Third, as some patients with OSAS were diagnosed during treatment for CHD, this may have resulted in an overestimation of the actual risk of CHD in the OSAS group. Despite these limitations, this study may provide a basis for future research on the causal relationship between OS and cardiovascular diseases, promoting risk stratification and the management of these patients.

## Conclusion

In conclusion, patients with OS had deteriorating baseline characteristics and a higher prevalence of cardiovascular diseases, including heart failure and PH, than patients with COPD or OSAS. Moreover, OS patients were found to be at a higher risk for all-cause death during the 1-year follow-up, which might be associated with the CCI score, hypertension, PH and heart failure. Clinicians should be aware that patients with OS have a heavier burden of cardiovascular diseases, and early identification and appropriate treatment are of great importance for the management of these patients.

## Data Availability

The raw data supporting the conclusions of this article will be made available by the authors, without undue reservation.
